# Thioredoxin 1 is associated with the proliferation and apoptosis of rheumatoid arthritis fibroblast-like synoviocytes

**DOI:** 10.1007/s10067-017-3832-1

**Published:** 2017-09-15

**Authors:** Tianbao Lu, Ming Zong, Shasha Fan, Ying Lu, Shanhan Yu, Lieying Fan

**Affiliations:** Department of Clinical Laboratory, Shanghai East Hospital, Tongji University School of Medicine, No. 150, Jimo Road, Shanghai, 200120 People’s Republic of China

**Keywords:** Apoptosis, Fibroblast-like synoviocytes, Proliferation, Rheumatoid arthritis, Thioredoxin 1

## Abstract

We aimed to investigate the possible effects of thioredoxin 1 (Trx1) on the proliferation and apoptosis of rheumatoid arthritis fibroblast-like synoviocytes (RA-FLSs) and elucidate the possible mechanisms involved. We investigated the distribution and expression of Trx1 in synovial tissues from RA and osteoarthritis (OA) patients by immunohistochemistry and real-time polymerase chain reaction (RT-PCR) analyses. RA-FLSs were isolated and cultured under normoxic (21% oxygen) or hypoxic (3% oxygen) concentrations. Transfection of Trx1-siRNAs and a Trx1 overexpression construct was conducted to manipulate the expression of Trx1. Protein expression was detected by Western blot. Doxorubicin (Adriamycin, ADR) was used to induce apoptosis. LY-294002 was used for the inhibition of PI3K-Akt. Cell proliferation and apoptosis were determined by MTS (3-[4,5-dimethylthiazol-2-yl]-5-[3-carboxymethoxyphenyl]-2-[4-sulfophenyl]-2H-tetrazolium, inner salt) assay and flow cytometry, respectively. The mRNA and protein expression of Trx1 in RA tissues was higher than that in OA tissues. The expression levels of Trx1 and cell proliferation in RA-FLSs were increased under hypoxia in comparison to those under normoxia. In hypoxia, downregulation of Trx1 significantly suppressed FLS proliferation, and the expression of PI3Kp85, phospho-Akt, and Bcl-2, while notably increased FLS apoptosis and the expression of active Caspase3 and Bax. In normoxia, Trx1 overexpression promoted the FLS proliferation and the expression of PI3Kp85, phospho-Akt, and Bcl-2, but inhibited FLS apoptosis and the expression of active Caspase3 and Bax in FLSs. Such effects were partially repressed by LY-294002 treatment. Trx1 may play an important role in regulating the proliferation and apoptosis of RA-FLSs by modulating PI3K-Akt activation.

## Introduction

Rheumatoid arthritis (RA) is a chronic inflammatory disorder and characterized by chronic joint inflammation and synovial hyperplasia, which ultimately leads to progressive destruction of articular cartilage and bone. The synovial hyperplasia is featured by an overabundance of RA fibroblast-like synoviocytes (RA-FLSs). RA-FLSs not only display tumor-like destructive and invasive features but also affect the inflammatory microenvironment through secreting a variety of proinflammatory factors or interacting with immune cells [1, 2]. Therefore, RA-FLSs play an important role in the initiation and development of RA, and the therapy targeting RA-FLSs might improve clinical symptoms of inflammatory arthritis without inhibiting systemic immune responses [3].

Due to the enhanced proliferation of RA-FLSs, the oxygen consumption in RA synovium increases, which leads to synovial hypoxia and hypoperfusion [4]. In the 1970s, Lund-Olesen K et al. first revealed the hypoxic nature of RA synovium by measuring the oxygen tension in the synovial fluid of RA patients with a Clark-type electrode [5]. Subsequent studies have shown that synovial tissues in RA patients are hypoxic, with the oxygen tension of 2–4%, as compared with that of 9–12% in patients without RA [6–9]. The hypoxic condition observed in RA synovium is likely to activate a transcriptional response mainly driven by hypoxia-inducible factors (HIFs). Several signaling pathways responsible for RA-FLS proliferation have been proposed [2, 10], while the signaling pathways involved in RA-FLS proliferation under hypoxia are poorly understood. Hypoxia activates phosphatidylinositol 3-kinase (PI3K)-serine/threonine kinase (Akt) pathway [11, 12], which plays a pivotal role in cell proliferation, migration, invasion, and survival [13]. Recently, a study has reported that hypoxia-induced proliferation and invasion of RA-FLSs are mediated through the PI3K-Akt pathway, suggesting a critical role of this pathway in RA progression [12, 14].

Thioredoxin 1 (Trx1), a 12 kDa protein with redox-active dithiol in the highly conserved active site (Cys–Gly–Pro–Cys), is ubiquitously expressed in all tissues of the human body [15]. Trx1 is involved in redox-dependent signaling via regulating HIFs [16, 17] and PI3K-Akt pathway [18–20]. Trx1 levels have been found elevated in the synovial fluid, serum, and synovial tissues of RA patients [21–23]. Trx1 is known to be induced by hypoxia and by proinflammatory stimuli, both of which are features of RA synovia [22–24]. The roles of Trx1 overexpression in tumorigenesis have been widely investigated [24], while little is known about its function on hypoxia-induced RA-FLS proliferation. Our previous study based on proteomic analysis has found that the expression of Trx1 in RA-FLSs was higher than that in osteoarthritis (OA)-FLSs. The present study tested the hypothesis that Trx1 regulates the proliferation and apoptosis of RA-FLSs via PI3K-Akt pathway under normoxic (21% oxygen) or hypoxic (3% oxygen) conditions.

## Materials and methods

### Patients and controls

Synovial tissues were obtained from six patients with RA (two males, four females, mean age: 65 ± 9 years) and six patients with osteoarthritis (OA) (three males, three females, mean age: 62 ± 11 years) who underwent knee arthroscopic or replacement surgery at Shanghai East Hospital. The clinical characteristics of these patients were listed in Table 1. All patients fulfilled the diagnosis of the American College of Rheumatology for RA [25] and OA [26]. Informed consent was obtained from each of the enrolled patients, and the study protocol was approved by the Ethics Committee of Shanghai East Hospital.

**Table 1:** Clinical and serological characteristics of the patients

Patient no.	Diagnosis	Gender	Age (years)	Disease duration (year)	Medications taken	ESR (mm/h)	RF (IU/mL)	CRP (mg/L)	Anti-CCP (IU/L)
1	RA	Female	72	15	MTX + Leflunomide	24	48.0	62.4	26.71
2	RA	Male	48	8	MTX + SSZ	36	127.8	33.4	38.3
3	RA	Female	56	5	SSZ + Leflunomide	41	38.6	29.7	12.9
4	RA	Female	69	12	MTX + SSZ	29	71.6	72.5	35.8
5	RA	Female	59	6	MTX + Leflunomide	19	68.4	33.8	14.6
6	RA	Male	67	7	MTX + Leflunomide	51	112.2	48.6	35.8

*RA* rheumatoid arthritis, *MTX* methotrexate, *SSZ* sulfasalazine, *ESR* erythrocyte sedimentation rate, *RF* rheumatoid factor, *CRP* C-reactive protein, *anti-CCP* anti-cyclic citrullinated peptide antibody

### Immunohistochemistry for synovial tissue

All specimens were fixed in 10% neutral buffered formalin, embedded in paraffin, and cut into 5-μm-thick tissue sections. The tissue sections were deparaffinized and rehydrated for immunohistochemical staining. The sections were heated at 95 °C for 20 min with Dako Target Retrieval Solution (Dako, Copenhagen, Denmark). After blocking with 3% H_2_O_2_, the sections were incubated overnight with anti-Trx1 (1:200, no . 2285, Cell Signal Technology Inc., USA) at 4 °C. Following three rinses with phosphate-buffered saline (PBS), 15 min for each, the sections were incubated with secondary antibody (Envision™ Detection Kit, Dako) for 30 min at room temperature. Finally, after another three rinses with PBS, the sections were visualized by using diaminobenzidine substrate kit (Dako) according to the manufacturer’s instructions.

### Quantitative RT-PCR analysis

Total RNA was extracted from synovial tissues and FLSs using TRIzol™ (Invitrogen, Carlsbad, CA, USA), and reverse transcription was performed using first-strand cDNA Synthesis Kit (Takara, Dalian, China) according to the manufacturer’s instructions. Real-time polymerase chain reaction (RT-PCR) was performed using Premix Ex Taq SYBR Green PCR (Takara) on an ABI PRISM 7500 (Applied Biosystems, Foster City, CA, USA) according to the manufacturer’s instructions. The sequences of primers were used as follows: Trx1, forward 5′-AAGCCTTGGACGCTGCAG-3′, reverse 5′-CATCCTGACAGTCATCCACATCTACT-3′; GAPDH, forward 5′-TGACTTCAACAGCGACACCCA-3′, reverse 5′-CACCCTGTTGCTGTAGCCAAA-3′. GAPDH served as the internal control.

### Isolation and culture of RA-FLSs

Synovial tissues were immediately placed in Roswell Park Memorial Institute (RPMI) 1640 medium (Life Technologies, Carlsbad, CA, USA) and processed within 4 h. The tissues were minced and evenly spread on the bottom of cell culture flasks in RPMI 1640 medium at 37 °C for 6 h. Next, the tissues were incubated with RPMI 1640 medium supplemented with 10% fetal bovine serum and antibiotic-antimycotic solution (Invitrogen) at 37 °C in a humidified 5% CO_2_ atmosphere. Non-adherent tissue pieces were carefully removed by replacing the medium every 3 to 5 days and passaged when the primary FLSs reached 70–80% confluence. FLSs were grown further over four to eight passages. To characterize the cytological phenotype of synovial cultures, the third passage cells were stained with mouse monoclonal antibodies (mAb) against human CD14 and CD90 (9011-0149-025 and eBio5E10, respectively, eBioscience, San Diego, CA, USA) and the cells showed CD14 negative and CD90 positive as measured by flow cytometry (Beckman Coulter, Fullerton, CA, USA).

### Hypoxic conditions

To imitate the joint cavity hypoxic microenvironment of RA patients, hypoxia (3% O_2_) was induced by culturing cells inside a tri-gas incubator (Forma Scientific, Div. of Mallinckrodt, Inc., Marietta, Ohio) infused with a mixture of 3% O_2_, 5% CO_2_, and 92% N_2_ at 37 °C. The cells were cultured in a normal culture gas-mixture condition with 21% O_2_ component were served as control.

### Transfection of siRNA and plasmid

Predesigned specific small interfering RNA (siRNA) targeting human Trx1 mRNA (RefSeq NM_003329.3) (si-Trx1-1 and si-Trx1-2) and negative control siRNA (named NC) were synthesized by Genepharma Inc. (Shanghai, China). The targeting sequences as follows: si-Trx1-1, 5′-GUCAAAUGCAUGCCAACAUTT-3′; si-Trx1-2, 5′-CCACCAUUAAUGAAUUAGUTT-3′; NC, 5′-UUCUCCGAACGUGUCACGUTT-3′. The plasmid of pcDNA3.0-Trx1-Flag was purchased from Addgen (Teddington, UK). The transfection of siRNA and plasmid was performed using Lipofectamine® 2000 (Invitrogen, Carlsbad, CA, USA) following the manufacturer’s protocol.

### Cell proliferation assays

FLSs were plated at a density of 2 × 10^3^ cells/well in 96-well plates and cultured for different time periods. At the end of each time period, cell proliferation was determined by using the CellTiter 96® Aqueous One Solution Cell Proliferation Assay kit (Promega, USA) according to the manufacturer’s instructions. Briefly, 20 μl 3-(4,5-dimethylthiazol-2-yl)-5-(3-carboxymethoxyphenyl)-2-(4-sulfophenyl)-2H-tetrazolium, inner salt (MTS) was added to each well containing 100 μl medium and then incubated at 37 °C for 4 h. The absorbance was read at 490 nm on a spectrophotometric plate reader (Bio-Rad, Hercules, CA, USA) with a reference wavelength at 650 nm. Each assay was performed in quintuplicate, and all tests were repeated three times.

### Western blot analysis

The RA-FLSs were lysed in lysis buffer and centrifuged at 14,000 rpm for 15 min. The protein samples in the supernatant were immediately collected and the concentration was measured using the Bradford method (Bio-Rad, Hercules, CA, USA). Equal amounts of protein were separated by 10 or 15% SDS-PAGE and then transferred onto nitrocellulose membranes (Amersham Pharmacia Biotech, Uppsala, Sweden). After blocking with 5% skimmed milk in PBS containing 0.1% Tween-20 for 2 h at room temperature, the membranes were incubated with primary antibodies against Trx1, PI3Kp85 (no. 4292S), Akt (no. 9272), p-Akt (Ser473) (no. 4060S), Bcl-2 (no. 2870S) and Bax (no.m5023S) (all purchased from Cell Signal Technology Inc., USA), Caspase3 and active Caspase3 (ab32351 and ab13847 purchased from Abcam, Cambridge, MA, USA), and β-actin (sc-47778) (Santa Cruz Biotechnology Inc., USA), and subsequently incubated with secondary horseradish peroxidase antibody. The immunoreactive proteins were visualized using an enhanced chemiluminescent reagent (Millipore Corporation, USA).

### Flow cytometry analysis of apoptosis

The trypsinized cells were collected and cell apoptosis was determined using BD Pharmingen™ FITC Annexin V Apoptosis Detection Kit I (BD Biosciences, USA) according to the manufacturer’s instructions. The cells were washed twice with cold PBS and then resuspended in 500 μl 1 × binding buffer to a concentration of 1 × 10^6^ cells/mL. After adding 5 μl FITC Annexin V and 5 μl propidium iodide (PI), the cells were gently vortex and incubated at room temperature in the dark for 15 min and finally analyzed by a flow cytometer (Beckman Coulter, Fullerton, CA, USA).

### Statistical analysis

The experiments were done in triplicate in six different donors. Data were expressed as mean ± SD. Statistical analysis was done with the mean of the triplicates of each donor (*n* = 6). All statistical analysis was performed by using the statistical software SPSS 17.0 (SPSS, Inc., Chicago, USA). Paired samples were analyzed with *t* test. Block design was performed with Friedman M test. Two comparisons of multiple correlated samples were analyzed by *q* test. It was considered statistically significant when a *p* value was less than 0.05.

## Result

### Trx1 is abundant in RA synovial tissues

**Fig. 1:**
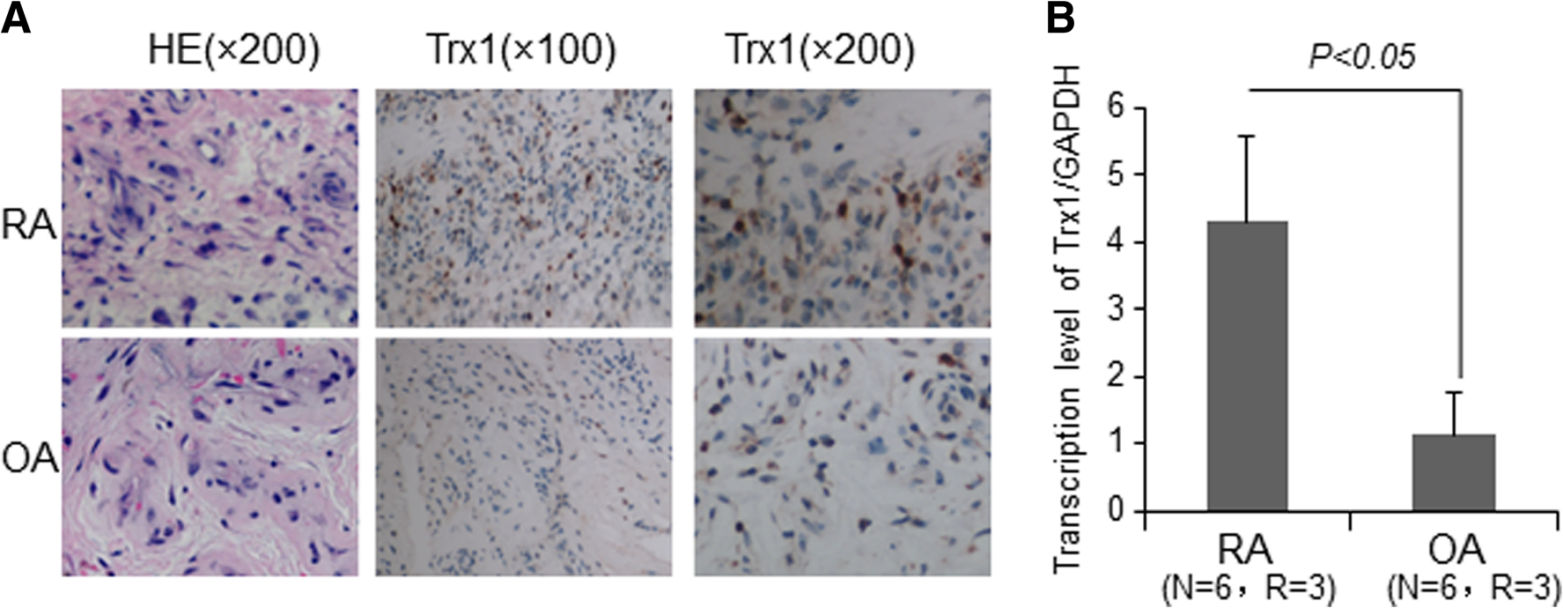
Trx1 is highly expressed in RA synovial tissues. **a** Immunohistochemical staining of Trx1 in synovium from RA and OA patients. **b** The mRNA levels of Trx1 in synovial tissues by quantitative RT-PCR. The data in **b** are means ± SD obtained from three separate experiments

To explore the distribution of Trx1 in RA and OA synovial tissues, we performed immunohistochemical staining. The results showed that Trx1 was expressed in both RA and OA synovial tissues. Trx1 was mainly localized in the cytosol and occasionally in the nucleus. Trx1 immunopositive cells were distributed diffusely (Fig. 1a). Furthermore, we detected the mRNA expression of Trx1 in synovial tissues by quantitative RT-PCR and found that Trx1 level was higher in RA synovial tissues than in OA (Fig. 1b). These results demonstrate that Trx1 is abundant in RA synovial tissues.

### Hypoxia promotes FLSs proliferation and induces Trx1 expression

**Fig. 2:**
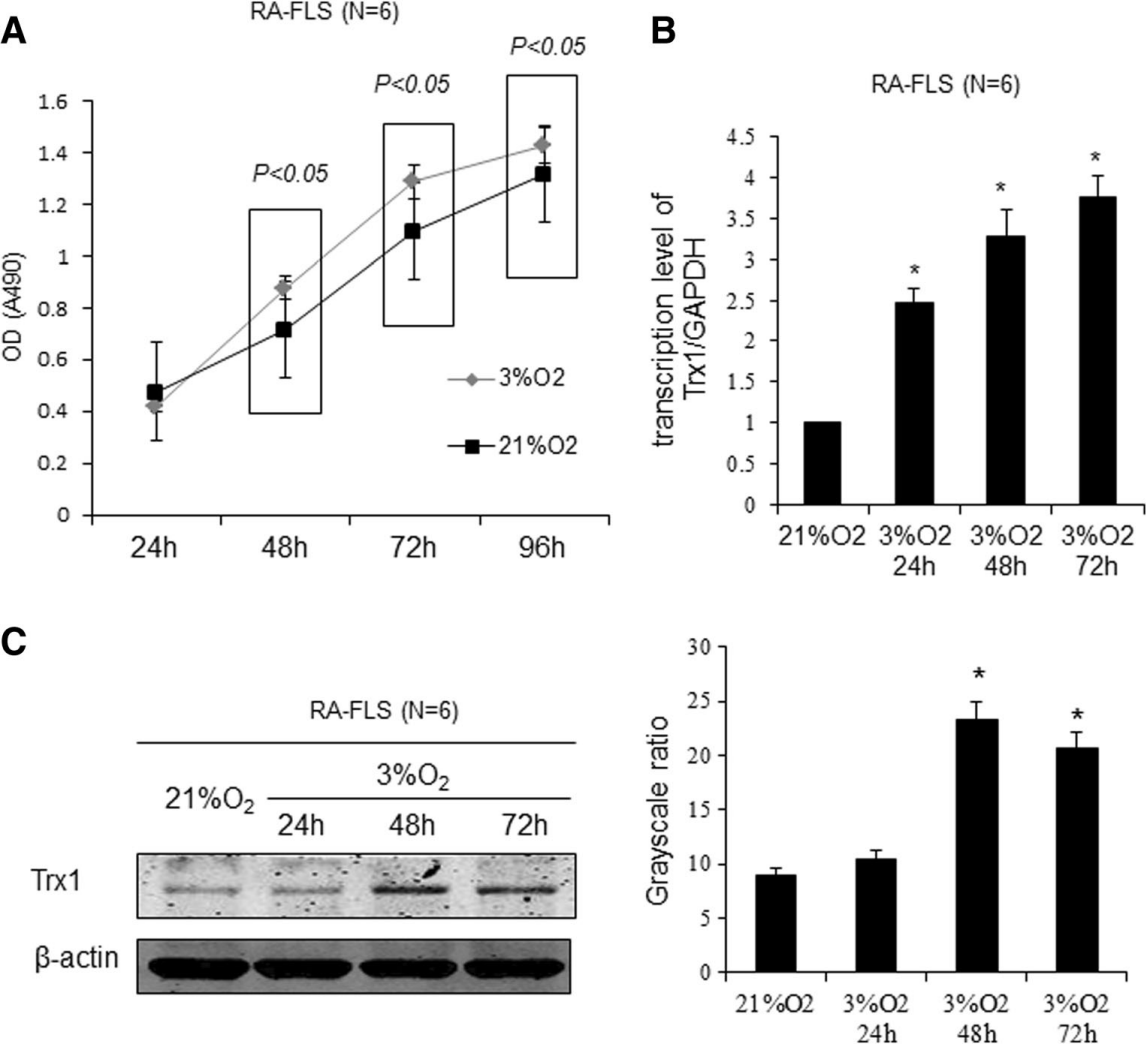
Hypoxia promotes RA-FLS proliferation and induces Trx1 expression in FLSs. **a** Effect of different oxygen concentrations on cell proliferation as assessed by MTS assay. **b** Trx1 mRNA expression levels were measured by quantitative RT-PCR analysis in RA-FLSs. **c** Trx1 protein expression levels were evaluated by Western blot analysis in RA-FLSs. The data are showed as means ± SD. (*P < 0.05 compared with the group that was cultured under 21% O_2_)

To investigate the effect of hypoxia on FLSs proliferation and Trx1 expression, RA-FLSs were cultured under conditions of different oxygen concentration (3% O_2_ and 21% O_2_) for different time periods. FLSs under hypoxia (3% O_2_) condition grew faster than those under normoxic (21% O_2_) (Fig. 2a). Subsequently, we analyzed the effect of hypoxia on the gene and protein expression of Trx1 in FLSs using quantitative RT-PCR (Fig. 2b) and Western blot (Fig. 2c), respectively. mRNA and protein levels of Trx1 were consistently upregulated in the hyperactive FLSs cultured under 3% O_2_ condition in a time-dependent manner as compared with those in FLSs under 21% O_2_.

### Trx1 affects the proliferation of RA-FLSs

**Fig. 3:**
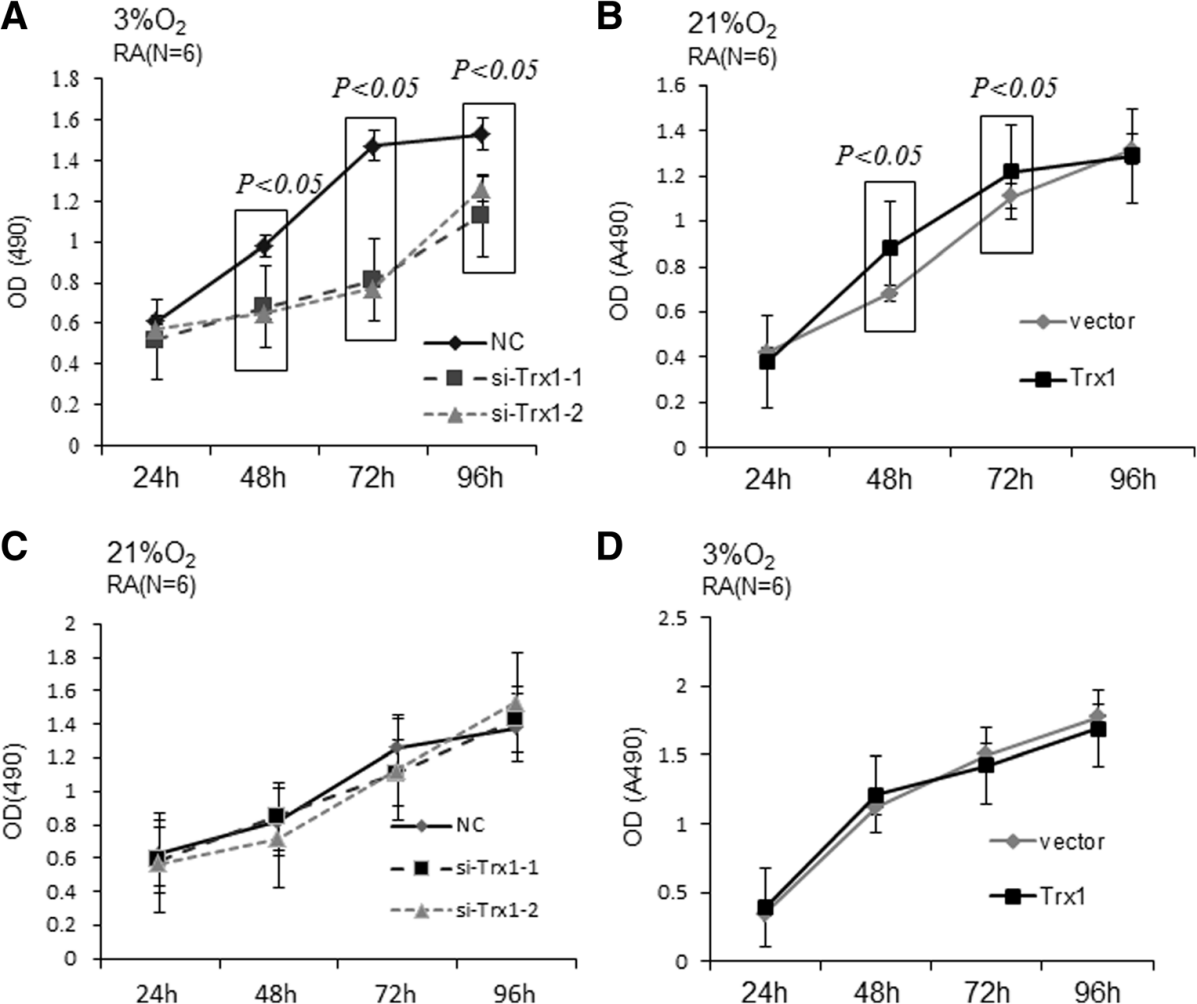
Trx1 affects the proliferation of RA-FLSs. **a**, **b** RA-FLSs were transfected with Trx1-siRNAs (si-Trx1-1 and si-Trx1-2) or control siRNA (NC) and cultured under 3% O_2_ (**a**) and 21% O_2_ (**b**) condition. The growth curve of RA-FLSs under 3% O_2_ condition was determined by MTS assay. **c**, **d** RA-FLSs were transfected with a Trx1 expression construct or control vector and cultured under 21% O_2_ condition. The growth curve of RA-FLS under 21% O_2_ (**c**) and 3% O_2_ (**d**) condition was assessed by MTS assay. The data are shown as means ± SD

To evaluate the effect of Trx1 on the proliferation of RA-FLSs, we modulated Trx1 expression levels in FLSs by transfection with Trx1-siRNAs (si-Trx1-1 and si-Trx1-2) or a Trx1 expression construct. The transfected cells were then cultured under hypoxia or normoxic condition. Trx1 knockdown effectively attenuated hypoxia-induced proliferation of FLSs at 48, 72, and 96 h after treatment as determined by MTS assay (Fig. 3a). Under normoxic condition, Trx1 knockdown showed no effects at other time points (Fig. 3b). On the contrary, Trx1 overexpression increased the proliferation of FLSs under normoxic condition (Fig. 3c) but had no effects on under hypoxia condition (Fig. 3d).

### Trx1 affects the apoptosis of RA-FLSs

**Fig. 4:**
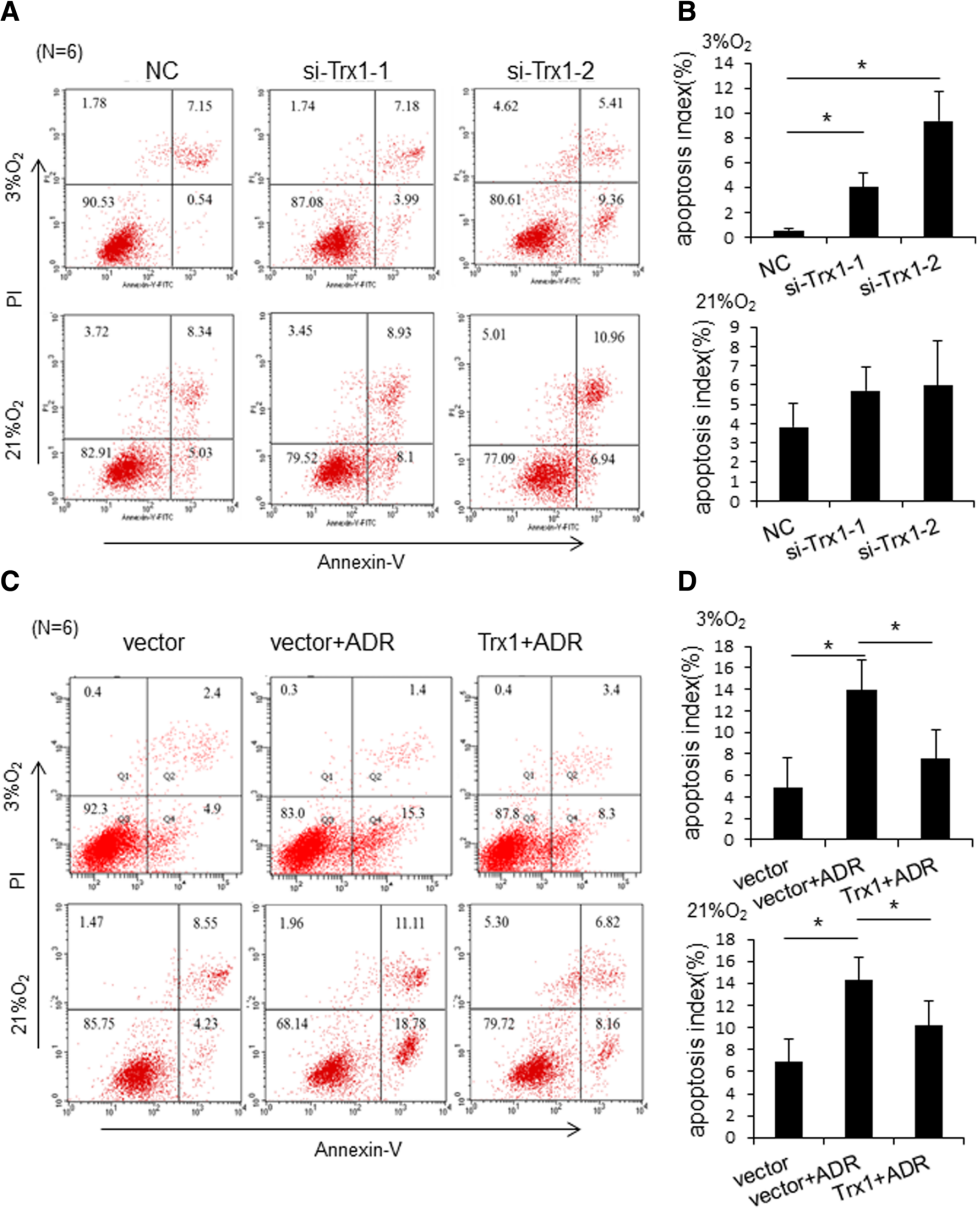
Trx1 affects the apoptosis of RA-FLSs. **a** RA-FLSs were transfected with Trx1-siRNAs (si-Trx1-1 and si-Trx1-2) or control siRNA (NC) and cultured for 48 h under 3% O_2_ and 21% O_2_ conditions. Cell apoptosis was then detected by flow cytometry. **b** Bar graph data of the early apoptotic cells (lower-right quadrant) are shown. **c** RA-FLSs were transfected with a Trx1 expression construct or control vector. After 48-h culture under 3% O_2_ and 21% O_2_ conditions, FLSs were incubated in the presence of 1 μg/mL of Doxorubicin (Adriamycin, ADR; Sangon, Shanghai, China) for 24 h. Cell apoptosis was then detected by flow cytometry. **d** Bar graph data of the early apoptotic cells (lower-right quadrant) are shown. The data in **a** and **c** are representatives of three separate experiments, and in **b** and **d** are shown as mean ± SD (*P < 0.05, **P < 0.01)

We then evaluated the effect of Trx1 on the apoptosis of RA-FLSs. Following transfection of Trx1-siRNAs, the percentages of early apoptotic FLSs were significantly increased under hypoxia condition and were not changed under normoxic condition (Fig. 4a, b). To further confirm the role of Trx1 in the apoptosis of FLSs, we used Adriamycin (ADR), a well-known apoptosis-inducing agent, to treat FLSs. Flow cytometry analysis showed that Trx1 overexpression diminished ADR-induced apoptosis of FLSs under both normoxic and hypoxia condition (Fig. 4c, d).

### Trx1 modulates PI3K-Akt activation and the expression apoptosis-related proteins in RA-FLSs

**Fig. 5:**
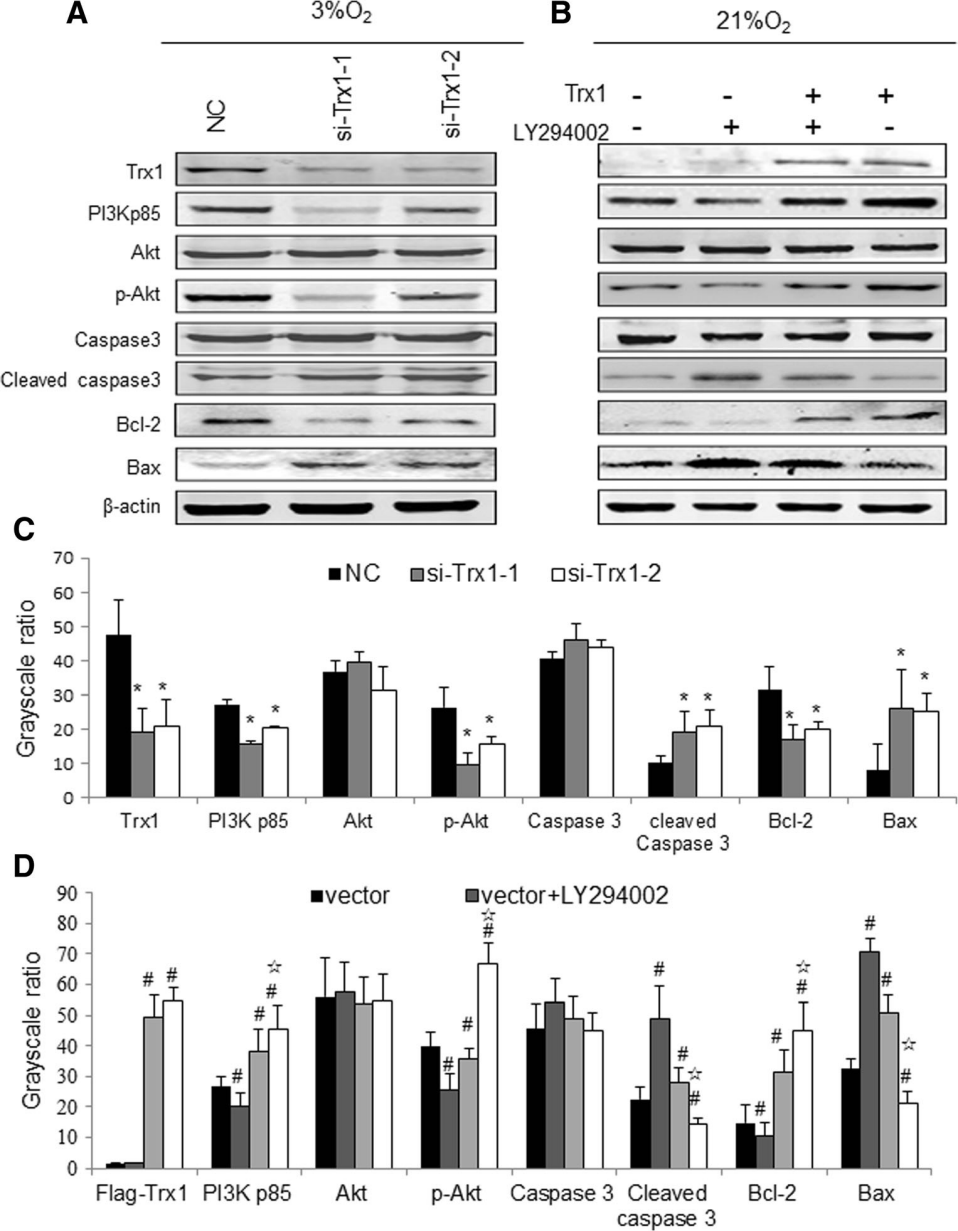
Trx1 modulates PI3K-Akt activation and the expression apoptosis-related proteins in RA-FLSs. **a**, **c** RA-FLSs were transfected with Trx1-siRNAs (si-Trx1-1 and si-Trx1-2) or control siRNA (NC) and cultured for 48 h under 3% O_2_ condition. Western blot and grayscale ratio analyses for the activation of PI3K-Akt and the expression of apoptosis-related proteins in RA-FLSs are shown. *P < 0.05. **b**, **d** RA-FLSs were transfected with a Trx1 expression construct or control vector. After 48-h culture under 21% O_2_ condition, 20 μM LY-294002 (Beyotime Biotechnology, Jiangsu, China) or an equal volume of DMSO was added to the cultured media. Cells were then incubated under normoxia for 24 h and Western blot analyses were then performed. Representative blot and grayscale ratio analyses of three separate experiments are shown (#*P* < 0.05 versus vector-transfected cells, ☆*P <* 0.05 Trx1-transfected cells versus Trx1-transfected and LY-294002-treated cell)

To elucidate the molecular mechanism underlying Trx1-attenuated apoptosis of RA-FLSs, the activation of PI3K-Akt and the expression of apoptosis-related proteins in RA-FLSs following incubation in hypoxia were detected. Trx1 knockdown significantly attenuated the protein level of PI3Kp85 and the phosphorylation of Akt at Ser473 in response to hypoxic exposure (Fig. 5A). Meanwhile, the apoptosis-related protein, Bcl-2, was also downregulated, but the active Caspase3 and Bax were upregulated by Trx1 knockdown in hypoxic FLSs (Fig. 5a).

To further confirm the role of Trx1 on the activation of PI3K-Akt, we overexpressed Trx1 in RA-FLSs under normoxic condition and assessed the levels of PI3Kp85, phospho-Akt, and apoptosis-related proteins. As shown in Fig. 5b, Trx1 overexpression evidently increased the levels of PI3Kp85, phospho-Akt, and Bcl-2 and decreased the levels of active Caspase3 and Bax under normoxic condition. In addition, the pharmacological PI3K inhibitor LY-294002 significantly repressed the effects of Trx1 overexpression on the expression of the above proteins.

## Discussion

FLSs from RA patients were found to display uncontrolled proliferation, which is causally related to the hypoxic microenvironment [27]. Our previous study has found that hypoxic exposure promotes the proliferation and reduces the apoptosis of RA-FLSs [28]. The increased level of Trx1 is closely correlated with the disease activity of RA, suggesting that Trx1 may become a useful clinical biomarker of RA [21–23]. However, little is known about the effect of Trx1 on the biological behavior of RA-FLSs.

Trx1 has been shown to regulate the proliferation and survival of tumor cells and prevent oxidative stress-induced apoptosis of tumor cells [29]. In our previous study, we found that the expression of Trx1 was higher in RA-FLSs than that in OA-FLSs [30]. In the present study, we found that the Trx1 levels were significantly increased in synovial tissues from RA patients compared with those in OA. In addition, we found that hypoxic condition significantly induced the proliferation of RA-FLSs and the Trx1 expression. These data suggest that hypoxia may be an inducer of Trx1 expression in RA-FLSs, which is intimately linked with the excessive proliferation.

One of the important pathophysiological characteristics of RA is synovial hyperplasia which leads to the destruction of articular cartilage and bone. The synovial hyperplasia is mainly due to an overabundance of FLSs. The overgrowth of FLSs in RA is largely from an imbalance between cell proliferation, survival, and death [1, 2]. In this study, we found that Trx1 knockdown effectively caused a significant decline in RA-FLS proliferation accompanied with increased cell apoptosis under hypoxia condition. However, Trx1 overexpression did not affect FLSs proliferation under hypoxia condition. It may be explained by that the increased Trx1 by hypoxia was sufficient to promote FLSs proliferation, and the ectopic overexpression of Trx1 had no addition effect. Meanwhile, Trx1 knockdown showed limited inhibitory effect on FLSs proliferation, but Trx1 overexpression significantly promoted the proliferation of RA-FLSs under normoxic condition. The possible cause of these findings was that RA-FLSs under normoxic condition had relative low expression of Trx1 and Trx1 knockdown could not further inhibit the proliferation of such cells. These results strongly imply that Trx1 is a key biological factor for the hyperproliferation and survival of RA-FLSs under hypoxia condition.

The PI3K-Akt pathway is abnormally activated in RA-FLSs, which inhibits cell apoptosis and promotes cell proliferation by affecting multiple downstream effector molecules [31]. Apoptosis is subtly regulated by anti- and pro-apoptotic molecules. Caspase3 is one of the most important proteases and plays a key role in the execution of apoptosis with other caspase cascades. Pro-apoptotic Bax and anti-apoptotic Bcl-2 are the most important members of Bcl-2 family which affect cell growth by modulating cell apoptosis. In this study, we found that knockdown of Trx1 expression inhibited the PI3K expression and Akt activation in RA-FLSs following incubation in hypoxia. Furthermore, we found that Trx1 knockdown caused a significant reduction in the expression of Bcl-2 but enhanced the expression of active Caspase3 and Bax in hypoxic RA-FLSs. These findings suggest that Trx1 inhibits RA-FLSs apoptosis by regulating activation of PI3K-Akt under hypoxic conditions. In normoxia, Trx1 overexpression evidently promoted the expression of PI3K and phospho-Akt in RA-FLSs. Moreover, we found that the upregulation of Trx1 markedly increased the expression of Bcl-2 and decreased the expressions of active Caspase3 and Bax under normoxic conditions. Such effects were partially suppressed by LY-294002, an inhibitor of PI3K. Our results also provide evidence that Trx1 and PI3K-Akt pathway may exert anti-apoptotic role under normoxic conditions.

In conclusion, our data strongly suggest that Trx1 may play a crucial pathophysiologic role in RA through the promotion of hyperproliferation of FLSs via a signaling pathway which depends on the activation of PI3K-Akt. Moreover, we have identified and demonstrated that Trx1 is highly expressed in RA-FLSs cultured under hypoxic conditions. Therefore, Trx1 may be a promising therapeutic target for RA.

## References

[1] Bottini N, Firestein GS (2012). Duality of fibroblast-like synoviocytes in RA: passive responders and imprinted aggressors. Nat Rev Rheumatol.

[2] Bartok B, Firestein GS (2010). Fibroblast-like synoviocytes: key effector cells in rheumatoid arthritis. Immunol Rev.

[3] Ganesan R, Rasool M (2017). Fibroblast-like synoviocytes-dependent effector molecules as a critical mediator for rheumatoid arthritis: current status and future directions. Int Rev Immunol.

[4] Muz B, Khan MN, Kiriakidis S, Paleolog EM (2009). Hypoxia. The role of hypoxia and HIF-dependent signalling events in rheumatoid arthritis. Arthritis Res Ther.

[5] Juranek I, Stern R, Soltes L (2014). Hyaluronan peroxidation is required for normal synovial function: an hypothesis. Med Hypotheses.

[6] Sivakumar B, Akhavani MA, Winlove CP, Taylor PC, Paleolog EM, Kang N (2008). Synovial hypoxia as a cause of tendon rupture in rheumatoid arthritis. J Hand Surg.

[7] Ng C, Biniecka M, Kennedy A, McCormick J, Fitzgerald O, Bresnihan B (2010). Synovial tissue hypoxia and inflammation in vivo. Ann Rheum Dis.

[8] Kennedy A, Ng CT, Biniecka M, Saber T, Taylor C, O'sullivan J (2010). Angiogenesis and blood vessel stability in inflammatory arthritis. Arthritis Rheum.

[9] Biniecka M, Kennedy A, Fearon U, Ng CT, Veale DJ, O’Sullivan JN (2009). Oxidative damage in synovial tissue is associated with in vivo hypoxic status in the arthritic joint. Ann Rheum Dis.

[10] Huber L, Distler O, Tarner I, Gay R, Gay S, Pap T (2006). Synovial fibroblasts: key players in rheumatoid arthritis. Rheumatology.

[11] Yan W, Fu Y, Tian D, Liao J, Liu M, Wang B (2009). PI3 kinase/Akt signaling mediates epithelial–mesenchymal transition in hypoxic hepatocellular carcinoma cells. Biochem Biophys Res Commun.

[12] Li GQ, Zhang Y, Liu D, Qian Y-Y, Zhang H, Guo SY (2013). PI3 kinase/Akt/HIF-1α pathway is associated with hypoxia-induced epithelial–mesenchymal transition in fibroblast-like synoviocytes of rheumatoid arthritis. Mol Cell Biochem.

[13] Carnero A (2010). The PKB/AKT pathway in cancer. Curr Pharm Des.

[14] Zhang Y, Zhang B, Trichostatin A (2015). An inhibitor of histone deacetylase, inhibits the viability and invasiveness of hypoxic rheumatoid arthritis fibroblast-like synoviocytes via PI3K/Akt signaling. J Biochem Mol Toxicol.

[15] Collet JF, Messens J (2010). Structure, function, and mechanism of thioredoxin proteins. Antioxid Redox Signal.

[16] Welsh SJ, Bellamy WT, Briehl MM, Powis G (2002). The redox protein thioredoxin-1 (Trx-1) increases hypoxia-inducible factor 1α protein expression. Cancer Res.

[17] Zhou J, Damdimopoulos AE, Spyrou G, Brüne B (2007). Thioredoxin 1 and thioredoxin 2 have opposed regulatory functions on hypoxia-inducible factor-1α. J Biol Chem.

[18] Sartelet H, Rougemont A-L, Fabre M, Castaing M, Duval M, Fetni R (2011). Activation of the phosphatidylinositol 3′-kinase/AKT pathway in neuroblastoma and its regulation by thioredoxin 1. Human Pathology.

[19] Meuillet EJ, Mahadevan D, Berggren M, Coon A, Powis G (2004). Thioredoxin-1 binds to the C2 domain of PTEN inhibiting PTEN’s lipid phosphatase activity and membrane binding: a mechanism for the functional loss of PTEN’s tumor suppressor activity. Arch Biochem Biophys.

[20] Chen B, Nelin VE, Locy ML, Jin Y, Tipple TE (2013). Thioredoxin-1 mediates hypoxia-induced pulmonary artery smooth muscle cell proliferation. Am J Phys Lung Cell Mol Phys.

[21] Jikimoto T, Nishikubo Y, Koshiba M, Kanagawa S, Morinobu S, Morinobu A (2002). Thioredoxin as a biomarker for oxidative stress in patients with rheumatoid arthritis. Mol Immunol.

[22] Heim K, Dälken B, Faust S, Rharbaoui F, Engling A, Wallmeier H (2016). High thioredoxin-1 levels in rheumatoid arthritis patients diminish binding and signalling of the monoclonal antibody Tregalizumab. Clin Transl Immunol.

[23] Hawkes HJ, Karlenius TC, Tonissen KF (2014). Regulation of the human thioredoxin gene promoter and its key substrates: a study of functional and putative regulatory elements. Biochim Biophys Acta.

[24] Burke-Gaffney A, Callister ME, Nakamura H (2005). Thioredoxin: friend or foe in human disease?. Trends Pharmacol Sci.

[25] Aletaha D, Neogi T, Silman AJ, Funovits J, Felson DT, Bingham CO (2010). Rheumatoid arthritis classification criteria: an American College of Rheumatology/European League Against Rheumatism collaborative initiative. Arthritis Rheum.

[26] Madry H, Kon E, Condello V, Peretti GM, Steinwachs M, Seil R et al (eds) (2016) Early osteoarthritis of the knee. Knee Surg Sports Traumatol Arthrosc 24(6):1753–1762 10.1007/s00167-016-4068-3 27000393

[27] Nonomura Y, Mizoguchi F, Suzuki A, Nanki T, Kato H, Miyasaka N (2009). Hypoxia-induced abrogation of contact-dependent inhibition of rheumatoid arthritis synovial fibroblast proliferation. J Rheumatol.

[28] Fan SS, Zong M, Zhang H, Lu Y, Lu TB, Fan LY (2015). Decreased expression of alpha-enolase inhibits the proliferation of hypoxia-induced rheumatoid arthritis fibroblasts-like synoviocytes. Mod Rheumatol.

[29] Kaimul AM, Nakamura H, Masutani H, Yodoi J (2007). Thioredoxin and thioredoxin-binding protein-2 in cancer and metabolic syndrome. Free Radic Biol Med.

[30] Zhang H, Fan L, Zong M, Sun L, Lu L (2012). Proteins related to the functions of fibroblast-like synoviocytes identified by proteomic analysis. Clin Exp Rheumatol.

[31] Bartok B, Boyle DL, Liu Y, Ren P, Ball ST, Bugbee WD (2012). PI3 kinase δ is a key regulator of synoviocyte function in rheumatoid arthritis. Am J Pathol.

